# MitophAging: Mitophagy in Aging and Disease

**DOI:** 10.3389/fcell.2020.00239

**Published:** 2020-04-15

**Authors:** Daniela Bakula, Morten Scheibye-Knudsen

**Affiliations:** Department of Cellular and Molecular Medicine, Center for Healthy Aging, University of Copenhagen, Copenhagen, Denmark

**Keywords:** autophagy, mitophagy, aging, mitophaging, monogenic disorders, interventions

## Abstract

Maintaining mitochondrial health is emerging as a keystone in aging and associated diseases. The selective degradation of mitochondria by mitophagy is of particular importance in keeping a pristine mitochondrial pool. Indeed, inherited monogenic diseases with defects in mitophagy display complex multisystem pathologies but particularly progressive neurodegeneration. Fortunately, therapies are being developed that target mitophagy allowing new hope for treatments for previously incurable diseases. Herein, we describe mitophagy and associated diseases, coin the term mitophaging and describe new small molecule interventions that target different steps in the mitophagic pathway. Consequently, several age-associated diseases may be treated by targeting mitophagy.

## Mitochondrial Integrity Defines Organismal Health

Mitochondria, the powerhouses of eukaryotic cells, are the key organelles for energy production allowing organismal growth and survival. Besides serving as adenosine triphosphate generators, mitochondria act as signaling hubs for programmed cell death, regulate calcium homeostasis and are required for cholesterol, nucleotide and amino acid synthesis (Sun et al., 2016). To fulfil their broad range of biological roles, mitochondria contain more than 1,000 proteins that localize and function in four specialized compartments, the outer membrane, the inner membrane, the intermembrane space and the matrix. The minority of mitochondrial proteins are encoded by the circular mitochondrial genome, whereas the vast majority is encoded in the nuclear genome. However, mutations in both genomes can cause a heterogeneous group of disorders, known as mitochondrial diseases, which are characterized by severe metabolic and neurological defects. Due to their highly variable clinical features, the prevalence of mitochondrial diseases has likely been underestimated (Haas et al., 2007; Wallace, 2018). Nevertheless, advances in next generation sequencing technologies have simplified the clinical diagnosis and enabled molecular characterization of so far undescribed mitochondrial diseases (Calvo et al., 2012; Cui et al., 2013; Legati et al., 2016). Notably, computational approaches relying on phenotypic description of mitochondrial diseases can help to characterize new mitochondrial diseases of previously unknown pathogenesis (Scheibye-Knudsen et al., 2013).

Increased evidence indicates that mitochondrial integrity is disrupted during the aging process and contributes to the pathogenesis of age-related disorders in humans (Kauppila et al., 2017; Youle, 2019). In line with this, mice that carry a defective proof-reading mitochondrial DNA polymerase gamma show an accelerated aging phenotype that may be driven by the accumulation of mutations in the mitochondrial DNA (mtDNA) (Trifunovic et al., 2004). The described correlation between levels of mtDNA deletions in human brain and aging as well as the association between mtDNA haplogroups and diseases, further supports the direct influence of mitochondria on health- and lifespan in organisms (Cortopassi and Arnheim, 1990; Corral-Debrinski et al., 1992; Hudson et al., 2014; Wallace, 2015). Indeed, dysfunctional degradation of mitochondria through the process of mitophagy is increasingly associated with degenerative diseases and aging, a phenomenon we call mitophaging. Evidently, the maintenance of functional mitochondria is necessary to sustain cellular homeostasis and organismal health.

## Mitochondrial Quality Control Mechanisms

Mitochondria have evolved multiple mechanisms ensuring mitochondrial quality. For instance, mitochondrial chaperones and proteases are constantly preventing the accumulation of misfolded and aggregated proteins by monitoring proteostasis through the mitochondrial unfolded protein stress response (UPR^mt^) (Melber and Haynes, 2018), a mechanism that has been shown to be critical for longevity in mammals (Houtkooper et al., 2013; Mouchiroud et al., 2013). Further, mitochondria are dynamic organelles existing in large tubular and highly dynamic networks that constantly undergo fission and fusion processes, thereby leading to the dilution of non-functional mitochondria (Youle and van der Bliek, 2012).

Nevertheless, autophagy is the only known pathway that mediates the turnover of whole mitochondria to avoid cellular damage and apoptosis. The degradation process is mediated by a double-membrane vesicle, known as the autophagosome, and it was first observed in mammalian cells by electron microscopy (De Duve and Wattiaux, 1966). For a long time, autophagy was considered a non-selective bulk degradation pathway, however, when the yeast mitochondrial protein Uth1p was found to be involved in the selective degradation of mitochondria (Kissová et al., 2004), the term “mitophagy” was subsequently introduced (Lemasters, 2005).

Herein, we discuss the role of mitophagy in impacting human disease development and the aging process itself. Further, interventions that target mitophagy will be discussed that may provide a promising strategy for the treatment of a broad spectrum of diseases.

## What Is Mitophagy?

**FIGURE 1:**
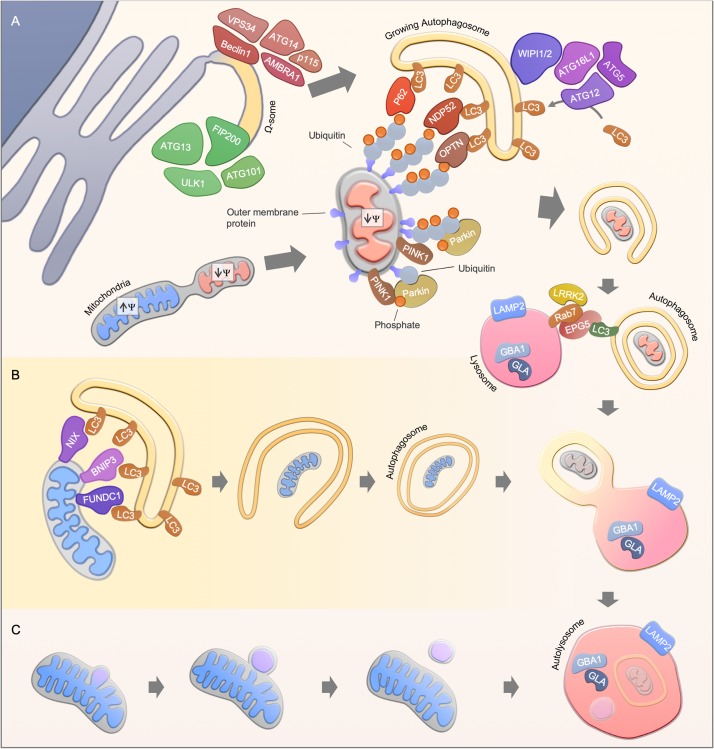
Mitophagy pathways. **(A)** Ubiquitin-dependent PINK1/Parkin-mediated mitophagy. Upon mitochondrial damage, PINK1 is stabilized at the outer mitochondrial membrane, leading to Parkin activation and subsequent ubiquitination of mitochondrial proteins. Finally, autophagy receptors such as NDP52, OPTN, and p62 are recruited to mediate the engulfment of mitochondria by the autophagosomal membrane through the interaction with LC3. A possible source of the autophagosomal membrane is provided by the endoplasmic reticulum, where the autophagy core complexes VPS34 and ULK1 initiate the membrane formation. The membrane formation is further mediated by WIPI1 and WIPI2, leading to the recruitment of the ATG16L1-complex and LC3, thereby facilitating the formation of autophagosomes. Finally, autophagosomes fuses with acidic lysosomes, a step that is regulated by concerted action of autophagosomal and lysosomal proteins. **(B)** Ubiquitin-independent receptor-mediated mitophagy. Ubiquitin-independent receptor mediated mitophagy is mediated by the recruitment of autophagy receptor proteins such as NIX, BNIP3, and FUNDC1 to the mitochondrial membrane. The receptor proteins recruit LC3, which enables the engulfment of mitochondria by autophagosomes. **(C)** Alternative degradation pathways. Piecemeal mitophagy and mitochondrial-derived vesicle degradation are cellular pathways that mediate localized degradation of mitochondria.

The process of mitophagy can act either as a response to various stress stimuli including nutrient starvation and oxidative stress or as a programmed removal of mitochondria (Palikaras et al., 2018; Pickles et al., 2018). Different pathways are known to regulate mitophagy, the best-studied pathway is mediated by the phosphatase and tensin homologue (PTEN)-induced putative kinase 1 (PINK1) and the E3-ubiquitin ligase Parkin (Figure 1A). Mutations in both genes encoding PINK1 and Parkin (PARK2), have been reported to cause autosomal recessive forms of Parkinson’s Disease (PD) (Kitada et al., 1998; Valente et al., 2004). Under un-stressed conditions, PINK1 is imported via the translocase of the outer membrane and translocase of the inner membrane (TOM/TIM) complex in a membrane potential dependent manner into mitochondria, leading to proteolytic cleavage of PINK1 (Jin et al., 2010; Deas et al., 2011; Meissner et al., 2011). The N-terminal truncated PINK1 is subsequently released to the cytosol, and degraded by the proteasome (Yamano and Youle, 2013). Loss of mitochondrial membrane potential disrupts the transport of PINK1 across the mitochondrial membrane leading to the accumulation of uncleaved PINK1 at the outer mitochondrial membrane. Subsequently, PINK1 regulates the recruitment and activation of the cytosolic Parkin via direct phosphorylation of the Parkin Ub-like (UBL) domain or via the phosphorylation of ubiquitin (Kondapalli et al., 2012; Shiba-Fukushima et al., 2012; Iguchi et al., 2013; Kane et al., 2014; Kazlauskaite et al., 2014; Koyano et al., 2014; Ordureau et al., 2014; Wauer et al., 2015). Once activated, Parkin drives the ubiquitination of multiple substrates, which leads to a positive feed forward mechanism through the generation of additional substrates for Pink1 (Ordureau et al., 2014).

In recent years, several substrates, in particular mitochondrial outer membrane proteins and autophagy receptors, have been identified to be ubiquitinated by the PINK1/Parkin-mediated signaling pathway (Sarraf et al., 2013). For instance, the mitochondrial fusion proteins mitofusin 1 and 2 (Mfn1 and Mfn2) are degraded in a PINK1/parkin dependent manner to make mitochondria accessible for degradation and to prevent fusion of damaged mitochondria with the healthy network (Gegg et al., 2010; Tanaka et al., 2010). However, conditional double-knockout of Mfn1 and Mfn2 in mice leads to mitochondrial dysfunction and, in line with this, Mfn2-depleted cardiomyocytes are deficient in Parkin recruitment to the mitochondrial outer membrane (Chen et al., 2011; Chen and Dorn, 2013). A similar priming function of mitochondria has been described for other mitochondrial proteins such as Miro1 and VDAC1 (Geisler et al., 2010; Wang et al., 2011; Sun et al., 2012; Safiulina et al., 2019). Recently, the apoptotic protein BAK has been identified as a Parkin target, further connecting Parkin-mediated mitophagy to the regulation of cellular apoptosis (Bernardini et al., 2019). The ubiquitination events driven by PINK1 and Parkin enable the recruitment of autophagy substrate receptors to the mitochondrial membrane including p62, Optineurin and NDP52, thereby promoting the engulfment of mitochondria by autophagosomes (Geisler et al., 2010; Wong and Holzbaur, 2014; Lazarou et al., 2015).

Notably, transcriptional regulation is a crucial process for functional PINK1-Parkin-mediated mitophagy. For instance, PINK1-Parkin-mediated mitophagy induction upon cellular stress such as through reactive oxygen species or ethanol exposure leads to the nuclear translocation of several transcription factors, including the transcription factor EB (TFEB) and the nuclear respiratory factors (NRFs), controlling the expression of mitochondrial, autophagy and lysosomal genes (Nezich et al., 2015; Ivankovic et al., 2016; Eid et al., 2019). Parkin expression itself has also been shown to be tightly controlled by stress pathways such as the unfolded protein response pathway and its activating transcription factor 4 (ATF4) (Bouman et al., 2011). Altogether, this highlights the great number of potential therapeutic avenues to target the PINK1-Parkin signaling pathway.

Pink1/Parkin-independent mitophagy pathways mainly rely on receptor proteins which mediate the recruitment of LC3/GABARAPs for the removal of mitochondria (Figure 1B). For instance, the BCL2-related protein NIX (also known as BNIP3L) mediates mitophagy in mammals during reticulocyte differentiation, a process that requires the elimination of mitochondria (Schweers et al., 2007; Sandoval et al., 2008; Novak et al., 2010). In line with this, *NIX* knockout mice develop anemia and reticulocytosis (Schweers et al., 2007; Sandoval et al., 2008). The interaction of NIX with LC3 protein members is mediated via the LC3-interacting (LIR) motif, however, re-expression of LIR-mutant NIX in NIX deficient reticulocytes partially rescued the observed phenotype, indicating LC3-independent or even autophagy-independent mechanisms for mitochondrial clearance in reticulocyte differentiation (Novak et al., 2010). Another LIR-motif containing protein, FUNDC1, regulates mitophagy under hypoxic conditions by promoting mitochondrial fission (Liu et al., 2012; Chen et al., 2016). During cardiac progenitor cell differentiation, FUNDC1 and NIX, but not Pink1 and Parkin, are upregulated to maintain a functional mitochondrial network (Lampert et al., 2019). Mitophagy is therefore also regulated in a lineage dependent fashion.

Localized removal of mitochondrial subdomains can be mediated by piecemeal mitophagy or mitochondrial-derived vesicles (Figure 1C). Mitochondrial-derived vesicle formation is thought to be dependent on PINK1/Parkin but independent of the canonical autophagy machinery (Soubannier et al., 2012; McLelland et al., 2014). Whereas, the accumulation of misfolded mitochondrial protein aggregates leads to localized recruitment of Parkin and autophagy proteins, thereby facilitating the degradation of mitochondrial subdomains (Burman et al., 2017). A PINK1/Parkin-independent piecemeal mitophagy has been recently reported that drives LC3C- and p62-mediated degradation of mitochondrial subregions (Le Guerroué et al., 2017). However, the protein machinery for these mitochondrial degradation pathways may overlap with the classic mitophagy pathways as well as their physiological relevance needs to be further investigated.

## Mitophaging

A decline in mitochondrial function is a hallmark of the aging process and is connected to other aging hallmarks such as telomere dysfunction, genome instability and cellular senescence. However, it remains largely unclear how these processes are interconnected and finally provoke disruption of the cellular and tissue integrity (López-Otín et al., 2013). There is accumulating evidence that mitophagy impacts health- and lifespan in different model organisms. Using a transgenic mouse strain that expresses the fluorescent mitophagy reporter mt-Keima, a decreased mitophagy level was observed in the hippocampal dentate gyrus in 21-month old mice compared to 3-month old mice (Sun et al., 2015). A decline in mitophagy was also observed in aged mouse hearts, in line with this, altered mitophagy has been shown to influence different cardiac pathologies (Hoshino et al., 2013; Bravo-San Pedro et al., 2017). Other tissues that contribute to aging phenotypes are also characterized by defective mitophagy, as shown recently for aged skeletal muscle satellite cells isolated from humans or mice (García-Prat et al., 2016). Notably, decreased expression of mitophagy genes was observed in the skeletal muscle of physically inactive elderly women (Drummond et al., 2014).

The effect of changes in mitophagy on health- and lifespan has been particularly demonstrated by using the model organisms *C. elegans* and *D. melanogaster*. Several genetic studies in *D. melanogaster* revealed that the overexpression of mitochondrial and mitophagy genes leads to increased health- and/or lifespan. For instance, the overexpression of the mitochondrial fission protein dynamin-related protein 1 (DRP1) increased the lifespan along with a prolonged healthspan in flies (Rana et al., 2017). The importance of mitochondrial fission on drosophila lifespan was further demonstrated by the observation that lifespan extension caused by the overexpression of p62 was abrogated in DRP1 mutant flies (Aparicio et al., 2019). Lifespan extension in flies was also observed after overexpression of Parkin and Pink1, whereby, Parkin overexpression counteracted increased Mfn2 levels, which can be observed during aging (Todd and Staveley, 2012; Rana et al., 2013). These findings are consistent with studies in *C. elegans*, where mitophagy has been shown to contribute to lifespan regulation (Palikaras et al., 2015; Schiavi et al., 2015). Evidently, there is substantial data supporting a role of declining mitophagy, mitophaging, in aging.

## What Happens When Mitophagy Goes Wrong?

**FIGURE 2:**
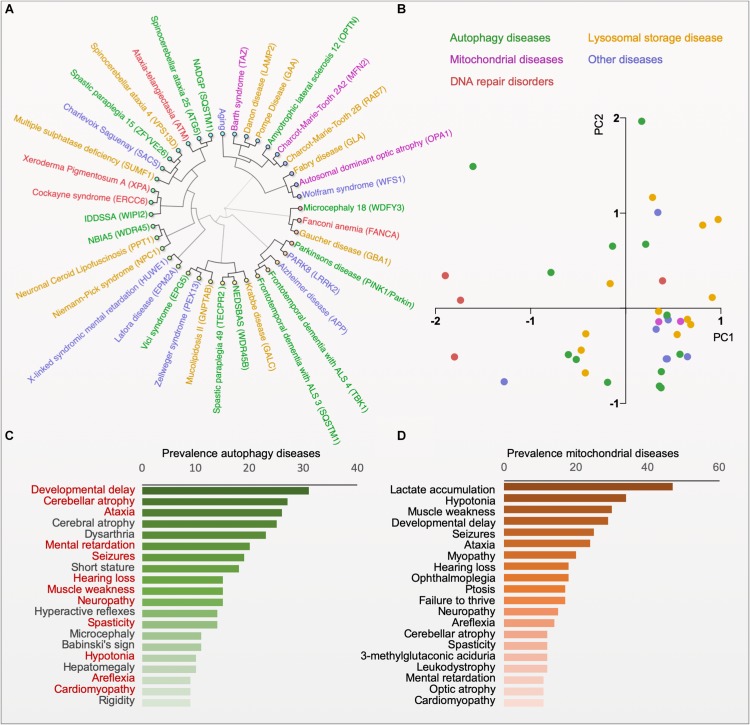
Phenotype clustering of autophagy diseases. **(A)** Hierarchical clustering of diseases based on the published prevalence of clinical features in the diseases (for data and references see www.mitodb.com). **(B)** Principal component analysis of diseases based on the prevalence of clinical features. **(C)** The average prevalence of top-20 clinical features in all autophagy-related disorders (Red, shared with the top-20 features in mitochondrial disorders). **(D)** The average prevalence of clinical features in mitochondrial diseases.

Impaired mitophagy contributes to the pathogenesis of several human diseases, in particular to age-related sporadic disorders, such as Parkinson’s disease, Alzheimer’s disease, cardiomyopathies and cancer (Bernardini et al., 2017; Fivenson et al., 2017; Levine and Kroemer, 2019). While these observations yield interesting correlations between certain disease states and alterations in mitophagy it is difficult to deduct causation. Here, monogenic diseases with specific defects in mitophagy may give us mechanistic understanding of pathogenesis and biology (Table 1). Thus, monogenic disorders may provide valuable tools for studying molecular pathomechanisms that are driven by defective mitophagy. To explore the clinical phenotype of autophagy diseases, we identified the clinical descriptions in the literature of all the diseases in Table 1 and performed hierarchical clustering based on the prevalence of those features (Figure 2A; Scheibye-Knudsen et al., 2013; Andreassen et al., 2019). Although the clustering did connect clinically similar diseases (such as Charcot–Marie–Tooth 2A2 and 2B), it became immediately apparent that there is no good correlation between clinical outcome and the putative molecular function of the gene responsible for the disease. Indeed, principal component analysis also did not show any obvious separation of clinical groups based on proposed molecular functions (Figure 2B). This suggests that our knowledge of the pathogeneses of most of these disorders is quite limited. Nevertheless, when looking at the average prevalence of clinical features across all aging diseases there was a considerable overrepresentation of neurological features suggesting that defects in autophagy often leads to brain disease (Figure 2C). More specifically, the phenotype in the autophagy disorders show significant overlap with what is seen in mitochondrial diseases indicating that mitochondrial dysfunction may be driving diseases in many autophagy-related disorders ranging from lysosomal diseases to bonafide mitophagy deficiencies (Figures 2C,D). In the following we will examine a few key examples of these disorders.

**TABLE 1:** Examples of autophagy/mitophagy-related monogenic disorders.

Disease	Gene	Protein function	Symptoms	References
Amyotrophic lateral sclerosis	OPTN (AD)	Autophagy receptor	Motor neuron degeneration	Weil et al., 2018
Alzheimer’s disease	APP (AD)	Transmembrane protein	Dementia	Fang et al., 2019
Ataxia-telangiectasia	ATM (AR)	DNA-damage response	Cerebellar degeneration, Telangiectasia, Radiosensitivity	Fang et al., 2016
Autosomal dominant optic atrophy	OPA1 (AD)	Mitochondrial fusion protein	Optic atrophy	White et al., 2009; Liao et al., 2017
Barth syndrome	TAZ (XLR)	Mitochondrial protein	3-Methylglutaconic aciduria, Cardiomyopathy, Neutropenia; Muscle weakness	Hsu et al., 2015
Charcot–Marie–Tooth disease	MFN2, RAB7 (AD, AR)	Mitochondrial fusion protein, endolysosomal protein	Neuropathy, Muscle weakness	Yamano et al., 2014; Rizzo et al., 2016
Charlevoix-Saguenay spastic ataxia	SACS (AR)*	Co-chaperone	Cerebellar degeneration, Neuropathy, Spasticity	Bradshaw et al., 2016; Morani et al., 2019
Cockayne syndrome	ERCC6 (AR)	DNA damage repair	Cerebellar degeneration, Short stature, Sun sensitivity	Scheibye-Knudsen et al., 2012
Danon disease	LAMP2 (XLD)	Autolysosome formation	Cardiomyopathy, Developmental delay, Myopathy	Tanaka et al., 2000; Hashem et al., 2017
Fabry disease	GLA (XL)	Lysosomal enzyme	Nephropathy, Cardiomyopathy, Hearing loss, Neuorpathy	Chévrier et al., 2010; Ivanova et al., 2019
Fanconi anemia	FANCC (AR)	DNA damage repair	Short stature, Anemia, Skin pigmentation changes, Osteopenia	Sumpter et al., 2016
Frontotemporal dementia and/or amyotrophic lateral sclerosis	TBK1, SQSTM1 (AD)	Serine/threonine protein kinase, autophagy receptor	Dementia, Motor neuron degeneration,	Geisler et al., 2010; Richter et al., 2016
Gaucher disease	GBA1 (AR)	Lysosomal enzyme	Hepatosplenomegali, Pancytopenia, Gaucher cells	Osellame et al., 2013
Intellectual developmental disorder with short stature and variable skeletal anomalies	WIPI2 (AR)	Autophagosome formation	Mental retardation, Cerebral atrophy, Short stature	Zachari et al., 2019
Krabbe disease	GALC (AR)*	Lysosomal enzyme	Spasticity, Leukodystrophy, Myoclonus	Del Grosso et al., 2019
Lafora disease	EPM2A (AR)	Glycogen synthesis	Seizures, Mental retardation	Lahuerta et al., 2018
Microcephaly 18	WDFY3 (AD)	Selective autophagy, aggrephagy	Cognitive deficits, Microcephaly	Napoli et al., 2018
MRXST	HUWE1 (XL)	E3-ubiquitin protein ligase	Mental retardation, Macrocephaly, Macroorchidism, Seizures	Di Rita et al., 2018
Mucolipidosis II	GNPTAB (AR)*	Lysosomal enzyme	Developmental delay, Short stature, Cardiomegaly, Dysostosis multiplex	Otomo et al., 2009
Multiple sulfatase deficiency	SUMF1 (AR)*	ER-resident enzyme	Cerebellar degeneration, Mental retardation, Hepatosplenomegaly	Settembre et al., 2008
NADGP	SQSTM1 (AR)	Autophagy receptor	Cerebellar degeneration, Mental retardation, Vertical gaze palsy, Dystonia	Geisler et al., 2010
NBIA5	WDR45 (XLD)*	Autophagosome formation	Cerebellar degeneration, Developmental delay, Brain iron accumulation, Dystonia	Saitsu et al., 2013
NEDSBAS	WDR45B (AR)*	Autophagosome formation	Seizures, Developmental delay, Spasticity, Cerebral atrophy	Bakula et al., 2017; Suleiman et al., 2018
Neuronal Ceroid Lipofuscinosis	PPT1 (AR)*	Lysosomal enzyme	Mental retardation, Seizures, Cerebellar degeneration	Mukherjee et al., 2019
Niemann-Pick disease	NPC1 (AR)*	Lysosomal protein	Seizures, Jaundice, Hepatosplenomegaly, Mental retardation	Pacheco et al., 2007
Parkinson’s disease	LRRK2, PARK2, PARK6 (AD)	Mitochondrial proteins	Bradykinesia, Rigidity, Tremor, Dementia	Ryan et al., 2015
Pompe disease	GAA (AR)*	Lysosomal enzyme	Muscle weakness, Cardiomyopathy, Hypotonia	Raben et al., 2012
Spastic paraplegia 15	ZFYVE26 (AR)*	Autophagosome formation	Spasticity, Hyperactive reflexes, Mental retardation	Vantaggiato et al., 2013; Denton et al., 2018
Spastic paraplegia 49	TECPR2 (AR)*	LC3/GABARAP binding protein	Developmental delay, Spasticity, Dysmorphism, Microcephaly, Hypotonia, Short stature	Oz-Levi et al., 2012
Spinocerebellar ataxia 25	ATG5 (AR)	Autophagosome formation	Developmental delay, Cerebellar degeneration, Mental retardation	Sun et al., 2015
Spinocerebellar ataxia 4	VPS13D (AR)	Lysosomal enzyme	Hyperactive reflexes, Muscle atrophy, Cerebeller degeneration	Anding et al., 2018
Vici syndrome	EPG5 (AR)*	Autolysosome formation	Cataracts, Cardiomyopathy, Developmental delay, Hypotonia, Immune deficiency, Corpus callosum agenesis	Cullup et al., 2013
Wolfram syndrome	WFS1 (AR)	Calcium homeostasis	Diabetes mellitus type 1, Optic atrophy, Hearing loss, Diabetes insipidus	Cagalinec et al., 2016
Xeroderma pigmentosum group A	XPA (AR)	DNA damage repair	Sun sensitivity, Cerebellar degeneration, Cancer, Neuropathy	Fang et al., 2014
Zellweger syndrome	PEX13 (AR)	Peroxisome biogenesis	Developmental delay, Dysmorphism, Hepatosplenomegaly, Seizures	Lee et al., 2017

*For genes that are marked with an asterisk the function in mitophagy remains largely unknown, however, defects in autophagy and mitochondrial dysfunction have been reported. Abbreviations: AD, autosomal dominant; AR, autosomal-recessive; XLR, X-linked recessive; XLD, X-linked dominant.*

### Defects in the Autophagic Machinery

To date, only a few monogenic diseases caused by single mutations in the autophagy core machinery have been reported. One of them, spinocerebellar ataxia-25 (SCAR25), is caused by a mutation in the *autophagy-related 5* gene (*ATG5)*, encoding a protein that is part of the ATG12-ATG5-ATG16L1 complex, which facilitates LC3/GABARAP conjugation (Mizushima, 2020). So far, two siblings have been identified with SCAR25, presenting with clinical symptoms such as truncal ataxia and intellectual disability (Kim et al., 2016). In line with the neurological phenotypes, a neuron-specific knockout of *ATG5* in mice causes neuronal degeneration, by contrast, a complete *ATG5* knockout is neonatal lethal (Kuma et al., 2004; Hara et al., 2006). Ataxia is a common feature of many mitochondrial disorders (Scheibye-Knudsen et al., 2013), however, mitochondrial viability in SCAR25 has not been investigated so far. Thus, the contribution of mitochondrial defects to the reported clinical features in SCAR25 remains speculative, since ATG5-independent mitophagy pathways have been reported (Honda et al., 2014; Hirota et al., 2015).

Mutations in members of the human WD-repeat protein interacting with phosphoinositides (WIPI) family are known to cause neurological deficits. The WIPI protein family consists of four members, WIPI1–WIPI4, that contribute to the early steps of autophagosome formation (Proikas-Cezanne et al., 2004). The family member WIPI2 localizes in a phosphatidylinositol 3-phosphate-dependent manner to the autophagosomal membrane, where it facilitates ATG16L1 recruitment and LC3 lipidation (Dooley et al., 2014; Bakula et al., 2017). Recently, patients with mutations in the *WIPI2* gene have been described with multisystemic clinical features, primarily, neurological and skeletal deficiencies that are characterized by severe mental retardation and short stature (Jelani et al., 2019). Notably, WIPI2 overexpression prevents age-related autophagy decline in dorsal root ganglion neurons (Stavoe et al., 2019). Patients with mutations in the genes *WIPI3* (*WDR45B*) or *WIPI4* (*WDR45*) show severe and progressive neurodegenerative phenotypes (Haack et al., 2012; Hayflick et al., 2013; Saitsu et al., 2013; Suleiman et al., 2018). Notably, *WIPI4* mutations result in degeneration of the substantia nigra, a target area of the brain affected in Parkinson’s disease (Mann et al., 1992). In line with these observations, *WIPI3* or *WIPI4* knockout mice show neurological defects, possibly caused by defective neuronal autophagy (Zhao et al., 2015; Ji et al., 2019). WIPI3 and −4 knockout mice display mitochondrial dysmorphology, which was also evident in WIPI4 mutant human fibroblast cells (Zhao et al., 2015; Seibler et al., 2018; Ji et al., 2019). The patient phenotypes caused by mutations in the *WIPI* genes highlight the importance of the WIPI protein members for neuronal function, however, the contribution of WIPI-mediated clearance of mitochondria in neurodegeneration remains unclear.

Deficiency in the late stage of autophagy is observed in the autosomal recessive neurological disorder, Vici syndrome. The disease is caused by mutations in the *ectopic P-granules autophagy protein 5* gene (*EPG5*), encoding for a Rab7 effector protein that is required for the fusion of late autophagosomes with lysosomes (Cullup et al., 2013; Wang et al., 2016). The disease is characterized by multisystemic defects that show some overlap with mitochondrial diseases, such as agenesis of corpus callosum, cardiomyopathy, immunodeficiency, cataracts and hypopigmentation (Cullup et al., 2013). Mitochondria with abnormal shape and distribution were observed in muscle tissue biopsies from patients with Vici syndrome or *EPG5* knockout mice (Cullup et al., 2013; Zhao et al., 2013). The importance of EPG5 in mitochondrial homeostasis was further highlighted by a study showing deficient mitochondrial clearance during spermatogenesis in an *EPG5*-deficient medaka fish line (Herpin et al., 2015).

Cargo recognition and degradation in selective autophagy is mediated by autophagy receptor proteins, such as optineurin and p62. Both proteins are associated with the progressive neurological disorder amyotrophic lateral sclerosis (ALS), which is primarily caused by loss of motor neurons (Maruyama et al., 2010; Fecto et al., 2011). Around 10% of ALS cases are caused by inherited single gene mutations and frequently show comorbidity with frontotemporal dementia (FTD). Interestingly, optineurin and p62 are phosphorylated by tank-binding kinase 1 (TBK1), a serine/threonine kinase that has also been implicated in ALS-FTD disease development (Cirulli et al., 2015; Freischmidt et al., 2015; Pottier et al., 2015). Thus, there is a striking correlation with mutations in multiple mitophagy players leading to ALS.

### Defects in Mitochondrial Quality Control

Proteins involved in the regulation of mitochondrial quality control are essential modulators of mitophagy, consequently, understanding their molecular mechanisms may give important insights into the consequences of impaired mitophagy. In recent years, mitochondrial dysfunction has been extensively discussed as an important contributor to neurodegeneration in familial Parkinson’s disease, as well as in idiopathic forms (Bose and Beal, 2016). Early onset recessive familial Parkinson’s disease can be caused by mutations in the genes *Park2* (*Parkin*), *Park6* (*Pink1*), or *Park7* (*DJ-1*). All three proteins localize to mitochondria and loss of each of them leads to increased sensitivity toward oxidative stress along with mitochondrial and energetic dysfunction (Dodson and Guo, 2007). Pink1 and Parkin are directly involved in the mitophagy pathway, whereas, the precise function of DJ-1 remains under discussion. Interestingly, overexpression of Pink1 and Parkin rescues the observed phenotype caused by DJ-1 deficiency, suggesting partial redundancies in the mitophagic apparatus (Irrcher et al., 2010).

Mitochondrial fission and fusion are critical events for controlled degradation of damaged mitochondria. Optic atrophy 1 (OPA1) is an inner mitochondrial membrane protein that regulates the fusion of mitochondria, together with MFN1 and MFN2. Mutation in the *OPA1* gene has been observed to cause autosomal dominant optic atrophy (ADOA) often accompanied by myopathy and progressive ataxia (Yu-Wai-Man et al., 2010). Myopathy and neurodegeneration is also observed in patients with Charcot–Marie–Tooth syndrome caused by loss of the *MFN2* gene (Calvo et al., 2009), underscoring the importance of mitochondrial function in muscle and brain tissues. For both diseases impaired mitophagy has been reported, suggesting that dysfunctional mitophagy may contribute to the described disease pathology (White et al., 2009; Rizzo et al., 2016; Liao et al., 2017).

### Defects in Lysosomal Function

Another group of diseases that may be partial driven by deficient mitophagy, are lysosomal storage disorders, a heterogenous group of more than 60 rare monogenic diseases that are caused by defects in lysosomal function (Platt et al., 2018). Some of the most well described are Gaucher disease and Niemann–Pick type C. Gaucher disease is caused by mutations in the *glucocerebrosidase* (*GBA*) gene, encoding a lysosomal enzyme required to hydrolyze the glycolipid glucosylceramide. Patients with Gaucher disease display features in multiple organs caused by lysosomal accumulation of glucosylceramide with a subset of patients display progressive neurodegeneration. Notably, the *GBA* gene represents a major risk locus for inherited Parkinson’s disease supporting the idea that mitophagy is important in this disease (Goker-Alpan et al., 2004; Lwin et al., 2004). Reduced mitochondrial respiration, increased ROS production and increased alpha-synuclein accumulation can be observed in various GBA deficiency models, cellular changes that are also described to be central drivers of neuronal loss in Parkinson’s disease (Osellame et al., 2013; Chen et al., 2019). Nieman Pick type C is caused by mutations in the *NPC1* gene and is characterized by developmental delay, progressive neurodegeneration, dysphagia and vertical gaze palsy, a combination of phenotypes that can also be observed in mitochondrial disorders. In patient-derived fibroblast cells and NPC1-deficient neuronal cells impaired autophagy and an accumulation of mitochondrial fragments have been observed upon lysosomal cholesterol accumulation (Pacheco et al., 2007; Elrick et al., 2012; Ordonez et al., 2012).

### Secondary Defects in Mitophagy

In addition to diseases with primary defects in mitophagy, several diseases have been described with secondary mitophagic dysfunction. In the context of monogenic diseases displaying premature aging, loss of mitophagy was first described in Cockayne syndrome, a disease characterized by progressive neurodegeneration reminiscent of mitochondrial disorders (Scheibye-Knudsen et al., 2012). The pathogenesis likely involves dysregulation of uncoupling proteins (U) due to decreased activity of the PGC-1alpha transcription factor. UCPs regulate mitochondrial membrane potential and consequently a reduction in UCPs lead to increased mitochondrial membrane potential and loss of PINK1 mediated mitophagy. Accordingly, overexpression of UCP2 can rescue mitochondrial and mitophagic defects in Cockayne syndrome. Notably, the same pathogenesis is found in related DNA repair disorders xeroderma pigmentosum group A and ataxia-telangiectasia (Fang et al., 2014, 2016).

Another disease that is characterized by mitochondrial deficiency is Zellweger syndrome, which belongs to a subgroup of peroxisome biogenesis disorders (Salpietro et al., 2015). Zellweger syndrome is caused by mutations in one of 14 human *PEX* genes, encoding for peroxin proteins that are required for the maintenance of peroxisomes (Waterham and Ebberink, 2012). Zellweger syndrome patients show dysmorphic features and suffer from severe neurological symptoms. Recently, PEX13 was shown to be required for mitophagy, but interestingly, dispensable for starvation-induced autophagy (Lee et al., 2017). Similarly, PEX5, an interaction partner of PEX13, has been shown to modulate autophagy via regulation of the mTOR signaling pathway (Eun et al., 2018), in line with this, mitochondrial defects can be observed in *PEX5* knockout models (Baumgart et al., 2001). However, it is still unclear, to what extent the clinical features of Zellweger syndrome are driven by mitophagic defects.

## Is Mitophagy a Therapeutic Target?

**FIGURE 3:**
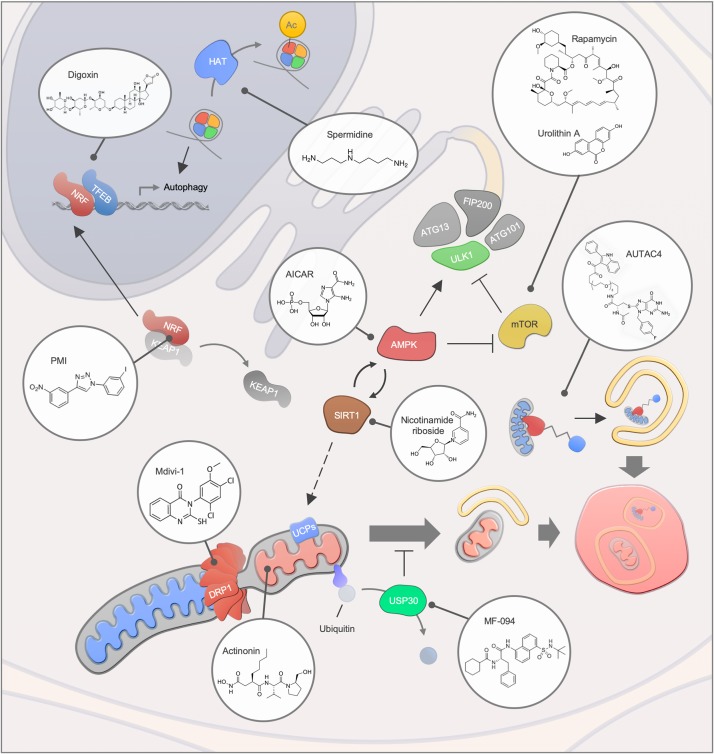
Mitophagy interventions. An overview of different mitophagy modulating compounds and their targets. Abbreviations: Ac, Acetylation; HAT, Histone acetyltransferase.

An increasing number of human diseases have been associated with impaired mitophagy, thus, interventions that modulate mitophagy may provide the possibility of counteracting disease development or progression (Figure 3). In recent years, multiple small molecules as well as lifestyle interventions have been shown to modulate autophagy, thereby causing health- and lifespan benefits in different organisms (Galluzzi et al., 2017). Due to the dependency on core autophagy regulators, mitophagy is modulated by most of the classic autophagy inducers such as the mTOR inhibitor rapamycin, the AMP-activated protein kinase (AMPK) activator AICAR as well as caloric restriction and exercise. In particular, the effectiveness of rapamycin and rapalogs has been intensively studied in the context of lifespan regulation and human disease development and rapamycin remain the most well documented compound for life- and healthspan extension in laboratory animals (Saxton and Sabatini, 2017). Further connections between longevity and mitophagy comes from work on the metabolite NAD^+^ and the NAD^+^-dependent acetylase Sirtuin 1 (SIRT1). Here, it has been shown that stimulation of SIRT1 through NAD^+^ augmentation or small molecules leads to activation of the energy responsive kinase AMPK that in turns regulates a central autophagy regulator, Unc-51-like kinase 1 (ULK1) (Egan et al., 2011; Price et al., 2012). Further, SIRT1 and AMPK also regulate the transcription factor PGC-1alpha, a key regulator of mitochondrial function that was initially found to control UCP levels and thereby mitochondrial membrane potential (Puigserver et al., 1998; Cantó et al., 2009). Indeed, SIRT1 activation leads to UCP-2 upregulation, stimulation of mitophagy and rescue of aging features in models of premature aging (Fang et al., 2014; Scheibye-Knudsen et al., 2014). Notably, direct stimulation of AMPK through the AMP-mimetic compound AICAR regulates mitochondrial dynamics via the induction of mitochondrial fission, further highlighting the broad effect of AMPK on mitochondrial function (Toyama et al., 2016).

Due to their great diversity, natural compounds are a tremendous source for novel mitophagy modulators. Urolithin A, a gut metabolite of ellagic acid, extends health- and lifespan in *C. elegans* as well as improving muscle function in rodent models via the induction of mitophagy (Ryu et al., 2016). The effectiveness of urolithin A was further highlighted in animal models of Alzheimer’s disease, where the disease pathology was ameliorated in the group of urolithin A-treated mice (Fang et al., 2019). In a human clinical trial study, the safety of urolithin A was evaluated, and signatures of improved mitochondrial function were demonstrated (Andreux et al., 2019). Similar to Urolithin A, the potency of antibacterial compound actinonin was demonstrated in Alzheimer’s disease models (Fang et al., 2019). Actinonin inhibits mitochondrial translation, thereby inducing mitophagy via the activation of the PINK1/Parkin-regulated signaling pathway (Richter et al., 2013; Sun et al., 2015; Burman et al., 2017). Another natural compound that has been suggested as a potential intervention for aging and diseases is the polyamine spermidine (Eisenberg et al., 2009; Madeo et al., 2018; Schwarz et al., 2018). The administration of spermidine leads to an induction of mitophagy in cardiomyocytes, along with cardio protection in mice (Eisenberg et al., 2016). The induction of autophagy via spermidine has been associated, among others, with the inhibition of the acetyltransferase EP300 and the ATM-driven activation of the PINK1/Parkin-regulated mitophagy pathway (Pietrocola et al., 2015; Qi et al., 2016).

Transcriptional regulation of mitophagy has also been shown as a viable pathway for increased mitochondrial health. An example is the synthetic compound PMI that stimulates mitophagy via the activation of the transcription factor Nrf2, which controls the expression of mitophagy genes including p62 (East et al., 2014; Bertrand et al., 2015). PMI treatment facilitates LC3 recruitment and mitochondrial ubiquitination in a PINK1/Parkin-independent manner, notably without disrupting the mitochondrial membrane potential (East et al., 2014).

Besides targeting mitophagy core proteins, intervention strategies targeting mitochondrial proteins may present a useful approach for disorders that are characterized by abnormal mitochondrial dynamic. Mdivi-1, has been identified in a yeast screen for mitochondrial fission inhibitors and several studies indicate its therapeutic potential for the treatment of neurological disorders (Cassidy-Stone et al., 2008; Cui et al., 2010; Solesio et al., 2012). However, the specificity of Mdivi-1 toward its putative target Drp1 has recently been questioned and needs to be further clarified (Bordt et al., 2017). USP30, a deubiquitinase that targets mitochondrial proteins, may present another promising target to facilitate mitophagy, since improved mitochondrial function was obtained upon USP30 depletion in different Parkinson’s disease models (Bingol et al., 2014). Notably, MF-094 has been recently identified as a selective inhibitor of USP30 that may thereby facilitate mitophagy through increased ubiquitination of outer membrane proteins (Kluge et al., 2018). Thus, a number of mitophagy modulators have been identified, yet the main goal will be the precise and specific targeting of damaged mitochondria. One possible way is to apply chimeric molecules such as the recently generated autophagy-targeting chimeric molecule (AUTAC4) that selectively targets the mitochondrial membrane for ubiquitination and subsequent degradation (Takahashi et al., 2019). These approaches may be particularly efficacious in conditions of mitophaging where the mitophagy apparatus is likely intact but mitophagy occurs at suboptimal levels.

In diseases characterized by dysfunctional lysosomes, stimulation of mitophagy may be detrimental due to an accumulation of undigested cargo material. In this regard, the inhibition of mitophagy is considered as a therapeutic strategy. In a mouse model of Pompe disease autophagy inhibition next to an enzyme replacement therapy has been proposed as a potential intervention (Raben et al., 2010). In line with this, knockdown of the mTOR pathway inhibitor TSC2 in muscle of Pompe disease mice reduced accumulation of autophagy markers and a decline in muscle atrophy was osberved (Lim et al., 2017). However, strategies to facilitate the fusion of autophagosomes and lysosomes in lysosomal storage disorders are also proposed for the treatment of several lysosomal storage disorders (Spampanato et al., 2013; Bartolomeo et al., 2017). TFEB, which controls the expression of autophagy as well as lysosomal genes and longevity (Napolitano and Ballabio, 2016), may provide a promising target since its agonists, such as the clinically approved cardiac drug digoxin or the natural compound ikarugamycin, improve metabolic function in mice and extend lifespan in *C. elegans* (Wang et al., 2017). The therapeutic potential of TFEB in Parkinson’s disease was further highlighted by a recent study that showed restored TFEB and improved neurological function upon rapamycin treatment in Q311X mutant parkin mice independently of the parkin E3 ligase (Siddiqui et al., 2015).

In summary, great progress has been made in recent years, however, the clinical safety of mitophagy modulating drugs needs to be further clarified. More refined tools that allow the distinction between mitophagy and general macroautophagy may be beneficial and could accelerate future discoveries. Altogether, this will enable us to step closer toward clinical validation of mitophagy modulators.

## Concluding Remarks

Mitophagy is emerging as a central process preserving organismal and, especially, neurological health. Since most trials targeting age-associated neurodegeneration in the last decades have been disappointing, new pharmaceutical avenues are direly needed. Here, mitophagy stimulators could play a key role. Indeed, several clinical trials are underway testing the efficacy of mitophagy modulating compounds and the outcome of these studies will undoubtedly prove critical for the future translatability of the field. Nonetheless, the regulatory mechanism of mitophagy and its contribution to age-associated diseases still remains elusive and potential issues with artificially augmenting mitophagy have not been considered. However, given the central role of mitophaging in multiple age-related pathologies it appears highly likely that these new promising approaches may present possible interventions in age-associated diseases. The future is bright!

## Author Contributions

DB and MS-K wrote the manuscript and made the figures.

## Conflict of Interest

The author declares that the research was conducted in the absence of any commercial or financial relationships that could be construed as a potential conflict of interest. The handling Editor declared a past collaboration with one of the authors, MS-K.
